# More than just blood, saliva, or sperm—setup of a workflow for body fluid identification by DNA methylation analysis

**DOI:** 10.1007/s00414-023-03069-z

**Published:** 2023-08-03

**Authors:** Helen Konrad, Leandra Jürgens, Benno Hartung, Micaela Poetsch

**Affiliations:** Institute of Legal Medicine, University Hospital Essen, Hufelandstr. 55, D-45122 Essen, Germany

**Keywords:** DNA methylation, Body fluid identification, Stain identification, Body fluids, Nasal secretion, Crime scene investigation

## Abstract

The determination of cellular origin of DNA is a useful method in forensic genetics and complements identification of the DNA donor by STR analysis, since it could provide helpful information for the reconstruction of crime scenes and verify or disprove the descriptions of involved people. There already exist several rapid/pre-tests for several secretions (blood, sperm secretion, saliva, and urine), RNA-based expression analyses (blood, menstrual blood, saliva, vaginal secretion, nasal secretion, and sperm secretion), or specific CpG methylation analyses (nasal blood, blood, saliva, vaginal secretion, nasal secretion, and sperm secretion) for determining the cell type.

To identify and to discriminate seven different body fluids and mixtures thereof in a simple workflow from each other, assays based on specific methylation patterns at several CpGs combined with pre-/rapid tests were set up in this study. For each of the seven secretions listed above, we selected the CpG marker achieving the highest possible discrimination (out of 30 markers tested). Validation studies confirmed a definite identification for saliva, vaginal secretion, and semen secretion in 100% of samples as well as discrimination from all other secretions. Moreover, the unambiguously correctly determined proportion of nasal samples, blood and menstrual blood varied between 61% (nasal blood) and 85% (nasal secretion).

In summary, our workflow proved to be an easy and useful tool in forensic analysis for the identification and discrimination of seven different body fluids often found at a crime scene.

**Supplementary Information:**

The online version contains supplementary material available at 10.1007/s00414-023-03069-z.

## Introduction

In addition to routinely performed short tandem repeat (STR) analysis to identify the DNA of victims or perpetrators or to establish relationships [1], more advanced analytical methods such as secretion identification are gaining importance in the context of forensic casework. The latter method provides additional information about the cellular origin of DNA and could thus contribute to the reconstruction of crime scenes [2, 3]. Especially in the context of sexual crimes, secretion analysis may prove or disprove those statements of involved people which could not be evaluated by proofing mere presence of victim’s or perpetrator’s DNA. For example, confirming the presence of vaginal secretions rather than epithelial cells could be crucial information.

In addition to the possibility of assessing a sample microscopically/histologically, there are enzymological or immunological test methods for some secretions based on the presence or absence of proteins [4]. However, these methods are not available for the identification of vaginal secretions or nasal secretions, are no longer applicable in the case of DNA that has already been extracted, e.g., in the context of cold cases, and allow only limited differentiation of nasal blood or menstrual blood from venous/arterial blood [5]. Since supplementary or new questions could always arise subsequently, RNA-based analytical methods are less suitable [4, 6], because ribonucleic acid is significantly less stable than DNA and degrades easily [7]. In contrast, DNA is a robust molecule that remains analyzable even after long periods of time and poor storage conditions [7, 8].

In 2011, Frumkin et al. [9] started to establish secretion-specific methylation assays in a forensic-genetic context and several others followed in the next years, so that specific assays have been developed for saliva, blood, vaginal secretions, menstrual blood, and semen [3, 10–15]. A good overview was published by Kader et al. in 2020 [16].

In a previous study, we presented specific CpGs for the identification of nasal secretions and nasal blood [17]. Moreover, we could show that the combination of pre/rapid tests with secretion-specific methylation analysis usually allows the identification and discrimination of nasal secretion, nasal blood, and semen against saliva, vaginal secretion, blood, and menstrual blood in a simple workflow [17].

This study aims to develop further methylation assays based on specific CpGs for additional secretions (blood, saliva, vaginal secretions, and menstrual blood) and thus to complete the workflow for identification and discrimination of body fluids by including them as well as rapid/pre-tests for peripheral blood, sperm secretion, and saliva. Our aim was to identify each of these secretions not only in single stains but also in mixtures of secretions typical of forensic issues.

## Material and methods

### Samples

The study was based on 415 samples of 155 adult individuals (age range 18–94 years) comprising 104 nasal samples (99 nasal mucosa and 5 nasal blood samples), 106 oral mucosa/saliva samples, 90 blood samples, 45 vaginal fluid samples, 41 menstruation blood samples, and 29 semen samples. Samples from each secretion were divided into 2 cohorts: the first one to establish the methylation assays and the second one for validation.

No information was available about diseases or operations like vasectomy or hysterectomy. Samples were collected between 2021 and 2023 in the Institute of Legal Medicine, University Hospital Essen, Germany.

### Compliance with ethical standards

All samples were obtained after informed consent and with approval of the Medical Ethics Committee at the University of Duisburg-Essen in accordance with the Declaration of Helsinki and national laws (ethic vote number: 21-9843-BO).

### Selection of CpG marker

For the identification or discrimination of different body secretions, CpG sites must have a unique methylation pattern. For the detection of nasal samples, the markers N21 and N27 have already been published in our previous study [17]. They were initially selected because they are sensitive to NOx and air pollution exposure [18]. To keep the naming and specificity assignment of all markers used in this study simple, these markers are renamed here as NB21 and N27SE. NB21 stands for specificity to nasal blood and N27SE for specificity to both nasal secretions and sperm secretions. Based on existing literature in the context of body fluid identification analysis, nine CpGs were selected for MSA or saliva (SA1–SA9) [3, 11–15], nine CpGs for blood (B1–9) [3, 11–15], six CpGs for vaginal secretion (V1–V6) [3, 11–14], and four CpGs for menstrual blood (MB1–MB4) [11–13]. Marker names, CpG IDs, and their associated genes including their function, chosen for analysis, are displayed in Table S1.

### Primer and assay design, DNA extraction, quantification, bisulfite conversion, amplification, and sequencing

Primer and assay design could be successfully performed for all markers using the PyroMark Assay Design 2.0 software (Qiagen, Hilden, Germany) and the PyroMark Q48 Autoprep software (Qiagen). DNA extraction was performed using the DNA IQ Casework Pro Kit and Casework Extraction Kit in the Maxwell® 16 instrument (Promega, Walldorf, Germany). Subsequently, DNA was quantified using the PowerQuant™ System (Promega) and converted using the MethylEdge Conversion System Kit (Promega). Pyrosequencing was done using PyroMark® PCR (Qiagen) and the PyroMark Q48 Autoprep instrument (Qiagen) plus using PyroMark® Q48 Advanced CpG Reagent Kit (Qiagen). All methods were performed as described in our previous study [17].

## Results and discussion

### Reliability of data

All samples used in this study were processed in exactly the same way. Therefore, an influence of the extraction methodology could be excluded. They showed a minimum concentration of 2.5 ng/μl, which results from the defined input amount of DNA into the bisulfite conversion. All bisulfite conversion controls during pyrosequencing were negative as desired, demonstrating successful conversion for all samples. Basically, all samples were analyzed as duplicates, and the deviation rate was a maximum of 5%.

### Body fluid identification

For an unambiguous and reproducible identification or discrimination of the different body secretions, reliable and meaningful DNA methylation markers are necessary. The aim is either a clear hypermethylation (> 90%) or a clear hypomethylation (< 10%) in the target fluid in contrast to the non-target [16]. To determine whether the results from the literature regarding the selected CpG markers and their respective specificity for saliva, blood, vaginal secretion, menstrual blood, or sperm secretion are reproducible, samples of each category were analyzed in all 28 CpG sites. However, for the majority of markers, no specific methylation range could be confirmed for the identification of the target secretion. This is not a contradiction to the literature results, but could be explained by the fact that not all seven different secretions were analyzed as sample material in the original studies. None of the previous studies analyzed nasal secretion or nasal blood [3, 11–15]; Park et al. [11] have not tested menstrual blood samples; Lee et al. and Forat et al. [12, 13] pooled vaginal secretion and menstrual blood samples and did not distinguish between these two secretions.

Based on our initial results, the best marker was selected for each type of secretion: NB21 (cg16518142) for nasal blood [17], B7 (cg13763232) for peripheral blood [15], MB4 (cg04255276) for menstrual blood [12], SA4 (cg21597595) for saliva [13], V2 (cg26079753) for vaginal secretion [3, 12], and N27SE (cg20864568) for nasal secretion and sperm secretion [17].

In these six remaining markers, DNA methylation percentage in the respective target secretion varied between 35 and 100% for nasal samples (NB21), 86 and 93% (plus seven outliers down to 60%) for peripheral blood (B7), 10 and 44% for menstrual blood (MB4), 17 and 60% for saliva (SA4), 31 and 78% for vaginal secretion (V7), 18 and 52% (plus one outlier down to 8%) for nasal samples (N27SE), and 87 and 95% (plus two outliers down to 81% and 73%) for sperm secretion (N27SE) (Table 1).

**Table 1:** Mean values and standard deviations for the selected CpG markers NB21, B7, MB4, SA4, V2, and N27SE including numbers of analyzed samples

	Nasal secretion/blood	Blood	Menstrual blood	Oral mucosa/saliva	Vaginal secreti**o**n	Sperm secretion
	(*n*)	(*n*)	(*n*)	(*n*)	(*n*)	(*n*)
	Mean value	Mean value	Mean value	Mean value	Mean value	Mean value
	Standard deviation	Standard deviation	Standard deviation	Standard deviation	Standard deviation	Standard deviation
NB_21 (cg16518142)	**46**	44	18	46	30	19
	**75.13**	89.93	82.94	91.96	82.23	98.21
	**17.55**	5.74	7.44	5.78	7.51	1.91
B_7 (cg13763232)	46	**42**	27	44	22	18
	37.65	**87.43**	41.37	30.57	30.91	9.06
	26.84	**7.11**	22.75	16.42	13.87	7.77
MB_4 (cg04255276)	44	47	**23**	45	24	18
	7.52	9.47	**20.39**	7.76	5.79	5.00
	4.49	3.53	**11.84**	3.68	3.11	2.69
SA_4 (cg21597595)	45	45	18	**46**	18	17
	7.49	5.82	5.22	**38.98**	6.89	2.00
	3.41	4.27	2.46	**10.91**	3.62	1.28
V_2 (cg26079753)	45	45	18	41	**22**	19
	8.51	12.60	22.33	11.32	**53.55**	8.89
	3.19	4.13	9.30	4.53	**13.35**	3.42
N_SE_27 (cg20864568)	**52**	46	20	42	21	**18**
	**31.40**	24.89	23.65	20.36	14.62	**90.39**
	**8.76**	3.97	8.82	5.80	3.85	**5.31**

Bold figures represent target secretion(s)

### Establishment of the workflow

**Fig. 1:**
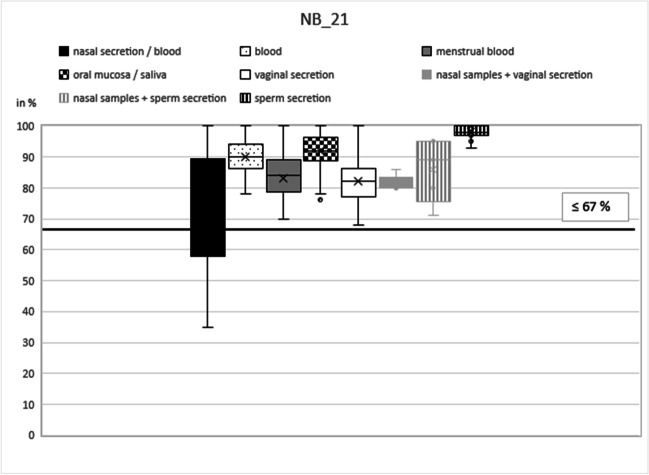
NB21 box plot. The box plots show the different methylation levels of the various body fluids for the respective CpG marker. The solid lines define cut-off values (without pre-tests); the dashed lines indicate threshold values under the condition of a negative pre-test results. These two critical values indicate the methylation range in which the target secretion could be clearly identified as such

**Fig. 2:**
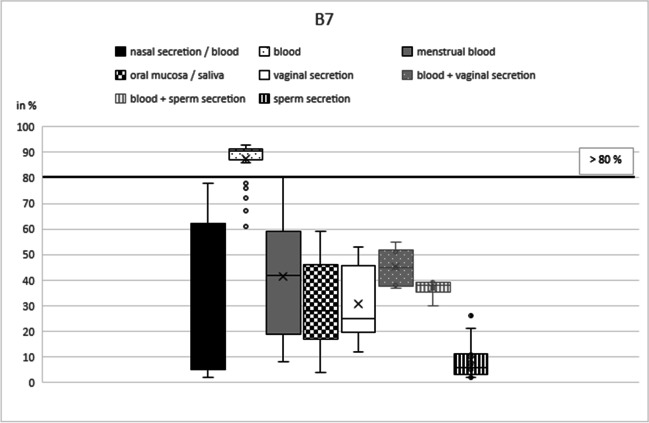
B7 box plot. The box plots show the different methylation levels of the various body fluids for the respective CpG marker. The solid lines define cut-off values (without pre-tests); the dashed lines indicate threshold values under the condition of a negative pre-test results. These two critical values indicate the methylation range in which the target secretion could be clearly identified as such

The next step in establishing the workflow was to determine a threshold for the identification of every secretion by testing the secretion-specific marker with the other body fluids. As in the previous study [17], the CpG marker NB21 showed the greatest variance for its target, nasal samples (mean 75%, standard deviation 18%; Table 1) (Fig. 1). Methylation results of blood, menstrual blood, saliva, vaginal secretion, and sperm secretion varied between 68 (vaginal secretion) and 100% (all other secretions). Therefore, the threshold value for NB21 from our previous study of 67% could be confirmed (Fig. 1, black line) [17]. Samples with a methylation value below 67% could thus be clearly identified as nasal blood or nasal secretions. Regarding the sample cohort for validation, this assay showed the worst result in relation to the target secretion and was able to identify only 14 of 23 samples as true positive for nasal samples (61%). There was no false-positive result for any kind of the other secretions (Table 2). Mixtures with nasal samples (nasal samples plus vaginal secretion and nasal samples plus sperm secretion) yield methylation values between 71 and 95% and could therefore not be identified as nasal sample mixtures (Fig. 2).

**Table 2:** Results of validation study

**NB21**	**n/nb in NB21**	**b in NB21**	**mb in NB21**	**sa in NB21**	**v in NB21**	**se in NB21**
**(*n*)**	**23**	**22**	**9**	**22**	**15**	**10**
True positive	**14**	-	-	-	-	-
In %	**60.87**	-	-	-	-	-
False positive	**-**	0	0	0	0	0
In %	**-**	0	0	0	0	0
True negative	**-**	22	9	22	15	10
In %	**-**	100	100	100	100	100
False negative	**9**	-	-	-	-	-
In %	**39.13**	-	-	-	-	-
**B7**	**n/nb in B7**	**b in B7**	**mb in B7**	**sa in B7**	**v in B7**	**se in B7**
**(*n*)**	**22**	**21**	**13**	**21**	**11**	**8**
True positive	-	**17**	-	-	-	-
In %	-	**80.95**	-	-	-	
False positive	0	**-**	0	0	0	0
In %	0	**-**	0	0	0	0
True negative	22	**-**	13	21	11	8
In %	100	**-**	100	100	100	100
False negative	-	**4**	-	-	-	-
In %	-	**19.05**	-	-	-	-
**MB4**	**n/nb in MB4**	**b in MB4**	**mb in MB4**	**sa in MB4**	**v in MB4**	**se in MB4**
**(*n*)**	**22**	**23**	**11**	**22**	**12**	**9**
True positive	-	-	**9**	-	-	-
In %	-	-	**81.82**	-	-	
False positive	0	0	**-**	0	0	0
In %	0	0	**-**	0	0	0
True negative	22	23	**-**	22	12	9
In %	100	100	**-**	100	100	100
False negative	-	-	**2**	-	-	-
In %	-	-	**18.18**	-	-	-
**SA4**	**n/nb in SA4**	**b in SA4**	**mb in SA4**	**sa in SA4**	**v in SA4**	**se in SA4**
**(*n*)**	**23**	**23**	**9**	**23**	**9**	**8**
True positive	-	-	-	**23**	-	-
In %	-	-	-	**100**	-	-
False positive	0	0	0	**-**	0	0
In %	0	0	0	**-**	0	0
True negative	23	23	9	**-**	9	8
In %	100	100	100	**-**	100	100
False negative	-	-	-	**0**	-	-
In %	-	-	-	**0**	-	-
**V2**	**n/nb in V2**	**b in V2**	**mb in V2**	**sa in V2**	**v in V2**	**se in V2**
**(*n*)**	**22**	**22**	**9**	**21**	**10**	**9**
True positive	-	-	-	-	**10**	-
In %	-	-	-	-	**100**	
False positive	0	0	0	0	**-**	0
In %	0	0	0	0	**-**	0
True negative	22	22	9	22	**-**	9
In %	100	100	100	100	**-**	100
False negative	-	-	-	-	**0**	-
In %	-	-	-	-	**0**	-
**N27SE**	**n/nb in N27SE**	**b in N27SE**	**mb in N27SE**	**sa in N27SE**	**v in N27SE**	**se in N27SE**
**(*n*)**	**27**	**23**	**10**	**21**	**10**	**8**
True positive	**23**	-	-	-	-	**8**
In %	**85.19**	-	-	-	-	**100**
False positive	**-**	0	0	0	0	**-**
In %	**-**	0	0	0	0	**-**
True negative	**-**	23	10	21	10	**-**
In %	**-**	100	100	100	100	**-**
False negative	**4**	-	-	-	-	**0**
In %	**14.81**	-	-	-	-	**0**

Results for target secretion(s) are displayed in bold

*n* nasal mucosa, *nb* nasal blood, *b* blood, *mb* menstrual blood, *sa* saliva, *v* vaginal secretion, *se* sperm secretion

The CpG marker B7 almost reached the hypermethylation cutoff (90%) for peripheral blood (mean 87%; standard deviation 7%; Table 1) (Fig. 2). The remaining secretions showed the greatest variance in this marker, with methylation percentages of 2% (nasal samples and sperm secretion) up to 80% (menstrual blood). There is no overlap between the target secretion and the others. A cutoff value could be set at 80% (Fig. 2, black line). Samples with a methylation value > 80% could be clearly identified as peripheral blood without any additional components. As expected, mixtures between peripheral blood and vaginal secretion could not be distinguished from menstrual blood. During validation, the marker identified 17 out of 21 samples correctly as true positive (81%). All other samples were found to be 100% true negative (Table 2).

**Fig. 3:**
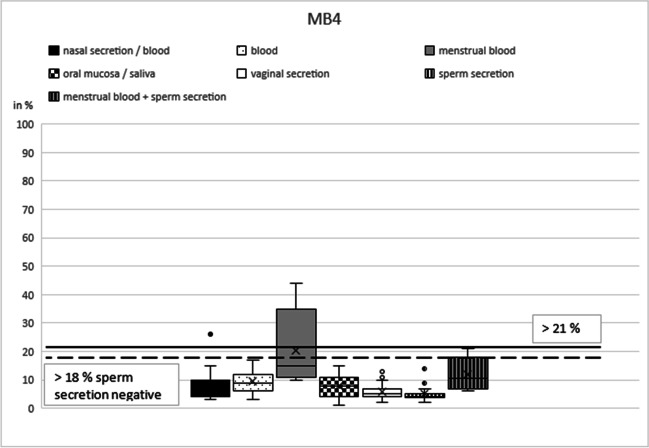
MB4 box plot. The box plots show the different methylation levels of the various body fluids for the respective CpG marker. The solid lines define cut-off values (without pre-tests); the dashed lines indicate threshold values under the condition of a negative pre-test results. These two critical values indicate the methylation range in which the target secretion could be clearly identified as such

The CpG marker MB4 showed an overlap between menstrual blood (mean 20%; standard deviation 12%; Table 1) (Fig. 3) and the other secretions. Methylation results of nasal blood, blood, saliva, vaginal secretion, sperm secretion, and nasal secretion varied between 1 (saliva) and 17% (blood). A cutoff line could be drawn at 21% (Fig. 3, black line), allowing clear identification of all menstrual blood samples that reach a methylation value > 21%. If the rapid test for sperm secretion is negative, the threshold could be lowered to > 18% (Fig. 3, dashed line). In case of a positive rapid test result for sperm secretion and a methylation value between 18 and 21%, a mixture of menstrual blood and sperm secretion could be assumed (Fig. 3, dashed line). In the validation study, nine of eleven samples (82%) could be determined as true positive. No sample was false positive (0%) (Table 2).

**Fig. 4:**
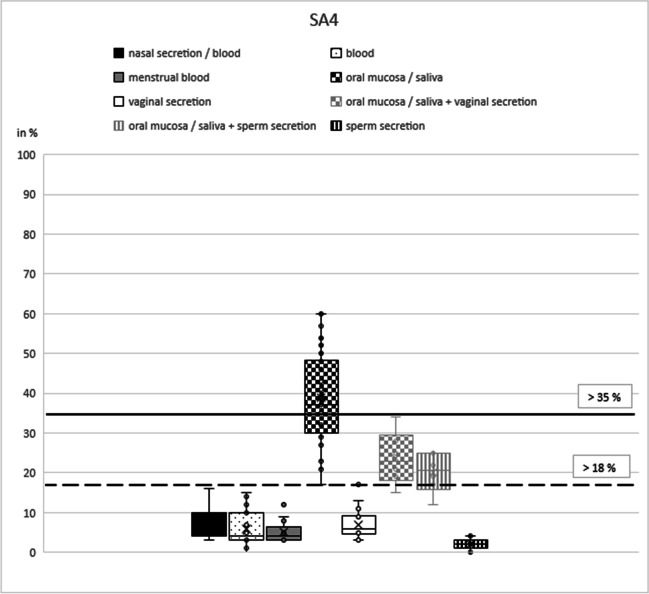
SA4 box plot. The box plots show the different methylation levels of the various body fluids for the respective CpG marker. The solid lines define cut-off values (without pre-tests); the dashed lines indicate threshold values under the condition of a negative pre-test results. These two critical values indicate the methylation range in which the target secretion could be clearly identified as such

The CpG marker SA4 showed almost complete hypomethylation for all secretions except saliva with methylation values between 0 (sperm secretion) and 16% (nasal samples). Saliva could be differentiated without overlap from other secretions (mean 39%; standard deviation 11%; Table 1) (Fig. 4). The threshold value at which a sample is clearly identified as saliva could be set at > 35% (Fig. 4, black line). A methylation value > 18% predicts saliva as a partial component of a mixture (Fig. 4, dashed line). Since the presence of blood cells and/or sperm secretion could be determined from the pre-test results, a mixture with vaginal secretion or nasal secretion (mixtures saliva and nasal secretions were not done) could be inferred in case of negative results of these pre-tests. The validation showed that all 23 samples could be correctly determined as true positive and all other samples as true negative under this assumption (Table 2).

**Fig. 5:**
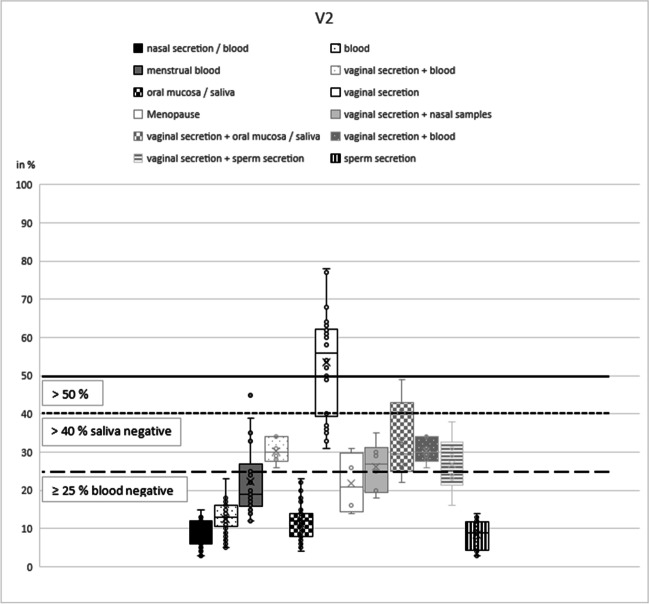
V2 box plot. The box plots show the different methylation levels of the various body fluids for the respective CpG marker. The solid lines define cut-off values (without pre-tests); the dashed lines indicate threshold values under the condition of a negative pre-test results. These two critical values indicate the methylation range in which the target secretion could be clearly identified as such

The CpG marker V2 showed an overlap between the methylation values of vaginal secretion (mean 54%; standard deviation 13%) and menstrual blood (mean 22%; standard deviation 9%; Table 1) (Fig. 5), so that a complete differentiation is not possible. At a methylation value of > 25%, the presence of vaginal tract secretions (vaginal secretion and menstrual blood) could be proved (Fig. 5, dashed line). This does not exclude a mixture of one or both secretions with others. A methylation value of > 40% and a negative saliva pre-test result unambiguously distinguish vaginal secretion from all other secretions, including menstrual blood (Fig. 5, dotted line). At a methylation level > 50%, vaginal secretions are clearly distinguishable from all other secretions independent of pre-test results (Fig. 5, black line). During validation, it was possible to identify nine out of ten (90%) vaginal secretion samples correctly with this marker (Table 2). Here, it is noteworthy that analysis of vaginal secretion samples from women after the onset of menopause (*n* = 4) showed a significant difference in the percentage of methylation (mean 22%; standard deviation 7%) (Fig. 5) compared to other vaginal secretion samples. Thus, the value resembles more that of saliva. However, more samples have to be analyzed before conclusions could be drawn.

**Fig. 6:**
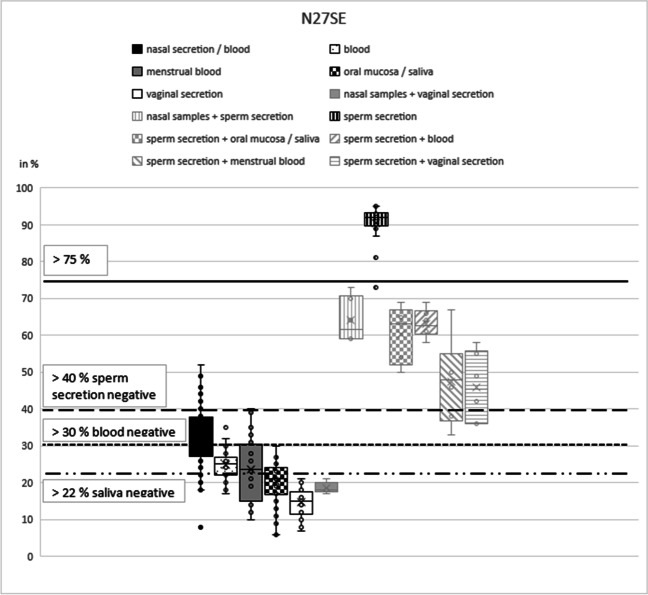
N27SE box plot. The box plots show the different methylation levels of the various body fluids for the respective CpG marker. The solid lines define cut-off values (without pre-tests); the dashed lines indicate threshold values under the condition of a negative pre-test results. These two critical values indicate the methylation range in which the target secretion could be clearly identified as such

As described before [17], the CpG marker N27SE shows a hypermethylation value for sperm secretion and the lowest standard deviation (mean 90%, standard deviation 5%; Table 1) (Fig. 6). Despite one downward outlier (73%), sperm secretion could be clearly distinguished from all other secretions at a methylation level of > 75% (Fig. 6, black line). This was confirmed during the validation process, which also showed that all eight sperm secretion samples could be identified as true positive (Table 2). Mixtures with sperm secretion reached methylation values between 33 (sperm secretion plus menstrual blood) and 73% (sperm secretion plus nasal samples).

Furthermore, this marker could identify a part of nasal samples (mean 31%, standard deviation 9%; Table 1) (Fig. 6) as described before [17]. The methylation value for nasal secretion overlaps to a large extent not only with menstrual blood (mean 25%, standard deviation 9%) but also with blood, saliva, and vaginal secretion. Nevertheless, under the assumption of a negative sperm secretion pre-test, the thresholds established in our first study could be confirmed [17]; at a methylation value > 40% and < 70%, nasal samples could be differentiated from the other secretions (Fig. 6, black and dashed line). Under the additional assumption of a negative blood pre-test, the lower threshold value decreased to > 30% (Fig. 6, dotted line); under the third assumption of a negative pre-test for saliva, the lower threshold value decreased to > 22% (Fig. 6, irregular line). A mixture of nasal secretion and vaginal secretion reached methylation values between 17 and 21% and could therefore not be identified as containing nasal secretion. Nevertheless, validation showed 23 of 27 (85%) nasal secretion samples could be correctly determined as true positive, there were no false-positive results for this marker (Table 2).

### Application of the workflow on unknown samples

**Fig. 7:**
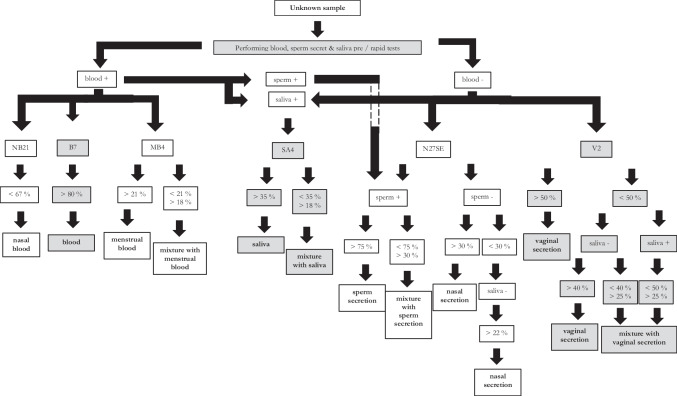
Workflow body fluid identification using pre-tests/rapid tests and methylation analysis; the procedure allows direct identification of seven different body fluids and mixtures thereof

In the following, a course of action is described for three unknown stains from casework according to the workflow (Fig. 7).

The first unknown trace was a light-colored application on a bed sheet. Preliminary tests for blood, saliva, and sperm secretions were performed. All three pre-tests showed a negative result; thus, these secretions could be excluded. A CpG analysis with this unknown sample was therefore performed with the markers V2, for the detection of vaginal secretions, and N27SE, for the detection of nasal secretions. Methylation analyses revealed a percentage of 8% for V2 and 29% for N27SE. The result for V2 showed vaginal secretion could not be detected. With a value of 29% in N27SE and the negative blood pre-test result, the cellular origin of the DNA of this unknown trace was clearly identified as nasal secretion.

The second unknown trace was a reddish application on a handkerchief. The three standard rapid tests yielded a positive result for both blood and seminal secretions, and a negative result for saliva. To determine which blood-containing secretion was present mixed with sperm secretion, methylation analysis was performed using the markers NB21, B7, MB4, and N27SE. It showed a percentage value of 90%, 38%, 20%, and 51% in NB21, B7, MB4, and N27SE, respectively. Based on the results of NB21 and B7, neither nasal blood nor peripheral blood could be detected. With a value of > 18% in MB4, menstrual blood is detected as a component of this mixture. The pre-test result and the result of N27SE with 51% identified sperm secretion as a sub-component of this trace. Accordingly, the bloody stains on the handkerchief are a mixture of menstrual blood and sperm secretion.

The last unknown trace was a towel with light-colored applications. Regarding standard pre-tests, blood and sperm tests showed negative results, saliva a positive one. The markers SA4, V2, and N27SE were selected for methylation analysis. A percentage of 28% could be determined for SA4, 43% for V2 and 17% for N27SE. The result for SA4 lead to the conclusion that saliva was present, at least as a partial component. Similarly, the V2 result verified a presence of vaginal secretion (at least as a partial component). The methylation value for N27SE disproved the presence of nasal secretion. Therefore, the light-colored staining on the towel consists of saliva and vaginal secretion.

## Conclusion

In this study, it was possible to set up, establish, and validate specific CpG marker and the corresponding methylation assays for seven different body fluids. The combination of highly specific and sensitive rapid/pre-tests for blood, saliva, and sperm secretion [33, 35 and 36] and secretion-specific methylation assays allowed the identification or differentiation of seven different body fluids and mixtures thereof (Fig. 7). Even if an unambiguous determination of 100% of the samples is not possible, the results obtained so far are applicable to legally relevant questions in most cases.

### Supplementary information

ESM 1

ESM 2
