# Supplementary material for: More than just blood, saliva, or sperm—setup of a workflow for body fluid identification by DNA methylation analysis
Source: Int J Legal Med. 2023 Aug 3;137(6):1683–92. doi: 10.1007/s00414-023-03069-z (PMC10567870; doi:10.1007/s00414-023-03069-z)
Supplement: Supplementary file 1 — ESM 1 [file 414_2023_3069_MOESM1_ESM.docx]

Table S1

| **marker** | **CpG ID** | **gene** | **function** | **published in context of body fluid identification:** |
| --- | --- | --- | --- | --- |
| NB_21 | cg16518142 | CDH26 | cell adhesion molecule, extracellular matrix | Konrad et al. (2023) [17] |
| N_27_SE | cg20864568 | MAP3K14 | signal transduction/ cell proliferation | Konrad et al. (2023) [17] |
| SA_1 | cg09652652 | FAM43A | lipoprotein receptor adaptor protein | Lee et al. (2015) [3]; Lee et al (2016) [12] |
| SA_2 | cg26107890 | SLC12A8 | cation-chloride cotransporter | Park et al. (2014) [11] |
| SA_3 | cg20691722 | SOX2-OT | important regulator of neurogenesis within long none coding RNA | Park et al. (2014) [11] |
| SA_4 | cg21597595 | unclassified | - | Forat et al. (2016) [13] |
| SA_5 | cg15227982 | WBP1L | WW domain binding protein 1-like | Forat et al. (2016) [13] |
| SA_6 | cg16732616 | DMRTA2 | transcription factor | Lin et al. (2016) [14] |
| SA_7 | cg09107912 | FNDC1 | fibronectin type III domain containing 1 | Lin et al. (2016) [14] |
| SA_8 | cg15731815 | RNF207 | RING finger protein 207 | Vidaki et al. (2016) [15] |
| SA_9 | cg08258650 | SLC1A2 | Excitatory amino acid transporter | Vidaki et al. (2016) [15] |
| B_1 | cg06379435 | unclassified | - | Park et al. (2014) [11]; Lee et al. (2015) [3]; Lee et al (2016) [12] |
| B_2 | cg01543184 | MAFG | bZip Maf transcription factor protein | Lee et al. (2015) [3] |
| B_3 | cg08792630 | unclassified | - | Park et al. (2014) [11]; Lee et al (2016) [12] |
| B_4 | cg26285698 | C16orf54 | transmembrane protein | Forat et al. (2016) [13]; Vidaki et al. (2016) [15] |
| B_5 | cg03363565 | RAB11FIP3 | Rab GTPase family / vesicles transport | Forat et al. (2016) [13] |
| B_6 | cg17518965 | S1PR4 | G protein-coupled receptor protein | Vidaki et al. (2016) [15] |
| B_7 | cg13763232 | SLC6A6 | Sodium- and chloride-dependent taurine transporter | Vidaki et al. (2016) [15] |
| B_8 | cg01607849 | TPI1 | Triosephosphate isomerase | Lin et al. (2016) [14] |
| B_9 | cg24124443 | BTBD11 | inhibitory interneuron specific synaptic scaffolding protein | Lin et al. (2016) [14] |
| V_1 | cg09765089 | unclassified | - | Lee et al. (2015) [3]; Lee et al 2016; Lin et al. (2016) [12] |
| V_2 | cg26079753 | unclassified | - | Lee et al. (2015) [3]; Lee et al (2016) [12] |
| V_3 | cg14991487 | HOXD9 | Homeobox protein, transcription factor | Forat et al. (2016) [13] |
| V_4 | cg03874199 | HOXD12 | Homeobox protein, transcription factor | Forat et al. (2016) [13] |
| V_5 | cg01774894 | HOXB3 | Homeobox protein, transcription factor | Park et al. (2014) [11] |
| V_6 | cg25416153 | unclassified | - | Lin et al. (2016) [14] |
| MB_1 | cg09696411 | SLC26A10 | multifunctional anion exchanger | Lee et al (2016) [12]; Forat et al. (2016) [13] |
| MB_2 | cg18069290 | SLC26A10 | multifunctional anion exchanger | Lee et al 2016 [12] |
| MB_3 | cg14991487 | HOXD9 | Homeobox protein, transcription factor | Park et al. 2014 [11] |
| MB_4 | cg04255276 | LTBP3 | Latent-transforming growth factor beta-binding protein 3 | Lee et al 2016 [12] |
