# Supplementary material for: More than just blood, saliva, or sperm—setup of a workflow for body fluid identification by DNA methylation analysis
Source: Int J Legal Med. 2023 Aug 3;137(6):1683–92. doi: 10.1007/s00414-023-03069-z (PMC10567870; doi:10.1007/s00414-023-03069-z)
Supplement: Supplementary file 2 — ESM 2 [file 414_2023_3069_MOESM2_ESM.docx]

Table S2

|  | **nasal samples + vaginal secretion** | **blood + vaginal secretion** | **oral mucosa / saliva + vaginal secretion** | **vaginal secretion + sperm secretion** | **nasal samples + sperm secretion** | **blood + sperm secretion** | **menstrual blood + sperm secretion** | **oral mucosa / saliva + sperm secretion** |
| --- | --- | --- | --- | --- | --- | --- | --- | --- |
|  | n | n | n | n | n | n | n | n |
|  | mean value | mean value | mean value | mean value | mean value | mean value | mean value | mean value |
|  | standard deviation | standard deviation | standard deviation | standard deviation | standard deviation | standard deviation | standard deviation | standard deviation |
| **NB_21** (cg16518142) | 5 |  |  |  | 5 |  |  |  |
|  | 81,60 |  |  |  | 86,00 |  |  |  |
|  | 2,24 |  |  |  | 9,30 |  |  |  |
| **B_7** (cg13763232) |  | 6 |  |  |  | 6 |  |  |
|  |  | 45,17 |  |  |  | 36,83 |  |  |
|  |  | 6,44 |  |  |  | 3,13 |  |  |
| **MB_4** (cg04255276) |  |  |  |  |  |  | 6 |  |
|  |  |  |  |  |  |  | 12,00 |  |
|  |  |  |  |  |  |  | 5,35 |  |
| **SA_4** (cg21597595) |  |  | 6 |  |  |  |  | 6 |
|  |  |  | 23,67 |  |  |  |  | 20,00 |
|  |  |  | 6,21 |  |  |  |  | 4,62 |
| **V_2** (cg26079753) | 6 | 6 | 6 | 6 |  |  |  |  |
|  | 26,17 | 30,33 | 32,83 | 26,83 |  |  |  |  |
|  | 5,87 | 2,98 | 9,37 | 6,91 |  |  |  |  |
| **N_SE_27** (cg20864568) | 5 |  |  | 6 | 6 | 6 | 6 | 5 |
|  | 18,60 |  |  | 46,00 | 64,00 | 63,17 | 47,50 | 60,20 |
|  | 1,36 |  |  | 8,66 | 5,66 | 3,62 | 10,81 | 7,08 |
